# Childhood sleep duration modifies the polygenic risk for obesity in youth through leptin pathway: the Beijing Child and Adolescent Metabolic Syndrome cohort study

**DOI:** 10.1038/s41366-019-0405-1

**Published:** 2019-07-08

**Authors:** Junling Fu, Yonghui Wang, Ge Li, Lanwen Han, Yu Li, Lujiao Li, Dan Feng, Yunpeng Wu, Xinhua Xiao, Mingyao Li, Struan F. A. Grant, Ming Li, Shan Gao

**Affiliations:** 1 Department of Endocrinology, NHC Key Laboratory of Endocrinology, Peking Union Medical College Hospital, Chinese Academy of Medical Sciences and Peking Union Medical College, 100730 Beijing, China; 2 Department of Endocrinology, Beijing Chaoyang Hospital, Capital Medical University, 100043 Beijing, China; 3 Department of Biostatistics and Epidemiology, University of Pennsylvania, Philadelphia, PA 19104 USA; 4 Division of Endocrinology, The Children’s Hospital of Philadelphia, Perelman School of Medicine, University of Pennsylvania, Philadelphia, PA 19104 USA; 5 Division of Human Genetics, The Children’s Hospital of Philadelphia Research Institute, Philadelphia, PA 19104 USA; 6 Department of Pediatrics, Perelman School of Medicine, University of Pennsylvania, Philadelphia, PA 19104 USA

**Keywords:** Obesity, Metabolic syndrome

## Abstract

**Background/objectives:**

Short sleep is an obesity risk factor, however, little is known about its interplay with genetic predisposition and pathways involved in obesity pathogenesis, especially in the longitudinal setting. We aimed to investigate a possible sleep-gene interaction for childhood obesity risk, and whether the interaction in childhood longitudinally contributes to obesity risk at a 10-year follow-up and further to test if there is any mediation through the leptin pathway.

**Subjects/methods:**

A total of 3211 children from China (6–18 years) at baseline and 848 participants at 10-year follow-up from the Beijing Child and Adolescent Metabolic Syndrome (BCAMS) cohort study were analyzed. Baseline leptin concentrations and 12 established adult body mass index (BMI) loci were examined for the associations with habitual sleep duration.

**Results:**

After adjusting for covariates, including pubertal stages and behavioral factors, short sleep duration at baseline was significantly associated with increased overweight/obesity risk at both baseline and follow-up. Genetic predisposition scores (GPS), particularly consisting of leptin-related SNPs (GPS_leptin_), were robustly associated with baseline overweight/obesity in children who slept ≤8 h/day (*P* *<* 0.001), whereas the association was ablated in those who slept ≥10 h/day (*P* *>* 0.05). Comparable observations were made at follow-up. Mediation analysis revealed a modest direct effect of the GPS_leptin_-sleep interaction on BMI at baseline, while a significant indirect effect of this interaction was found to be mediated principally through elevated leptin (proportion: 52.6%); moreover, the mediation effect via leptin remained stable over 10 years.

**Conclusions:**

This study suggests that shorter sleep duration in children from China (< 8h/day), compared to longer sleep duration (≥10 h/day), has a long-term impact on the association of polygenic risk for obesity from childhood to young adulthood and leptin pathway explains a key mechanism via a modification effect. Therefore, adequate sleep duration during childhood is important for the early prevention of obesity, especially if there is a genetic predisposition to this trait.

## Introduction

Obesity rates have increased sharply over the past 20–30 years, particularly in childhood. The epidemic of obesity poses a major risk for the development of type 2 diabetes, cardiovascular disease, and certain types of cancer^1^. It is now known that both genetic and environmental factors contribute to the complex etiology of this trait. Among the changeable environmental factors that can influence risk, the obesogenic diet, and low physical activity are considered two major adult risk factors; however, other potentially plausible environmental factors, like short sleep duration, have been gaining increasing attention in recent years [2, 3].

Meta-analysis of cross-sectional [4] and longitudinal studies [2] showed that short sleep duration was associated with the risk of obesity. However, the mechanistic links between sleep and obesity are poorly understood. Sleep duration can alter brain functions involved in the control of appetite, which can in turn lead to overeating in an obesogenic environment [5]. Meanwhile, the associations between sleep duration and appetite are likely to be mediated by multiple factors, including a change in the levels of the appetite-related hormone leptin evoked by sleep deprived [6]. Leptin, a hormone secreted by white adipocytes, acts on specific receptors in the hypothalamus, a key tissue in satiety regulation and day-night circadian, and thus has been recently proposed as one possible mechanistic link between decreased habitual sleep duration and increased risk of obesity in both adults [7] and children [8–12]. In our recent cohort study of children from China with risk for metabolic syndrome (MS) [13], we also found that short sleep duration was associated with both increased obesity risk and leptin concentrations. However, the studies concerning the association between leptin levels and sleep duration are still inconsistent [14] and the underlying mechanisms still need to be elucidated.

A number of genome-wide association studies have revealed compelling genetic signals influencing obesity risk both in adults and children [15]. Recently, we replicated the genetic associations of established East Asian adult body mass index (BMI) loci [16–18] with childhood obesity, and found that six previously identified brain-expressed obesity-related loci, namely *FTO*-rs1558902, *MC4R*-rs2331841, *MAP2K5*-rs4776970*, GNPDA2*-rs16858082, *PCSK1*-rs261967, and *BDNF*-rs2030323, exhibited significant association with both obesity risk and increasing leptin concentrations in children [19]. Importantly, previous studies have found that sleep duration could modify the effect of the *FTO* locus on both pediatric and adult BMI [20, 21]. Notably, other loci such as *MC4R, PCSK1*, and *BDNF*, are well known to be associated with severe early-onset obesity and harbor genes involved in the regulation of leptin-melanocortin pathways in the hypothalamus [15], thus are thought to affect body weight largely through impacting appetite. As such, these findings implicate both common variants and sleep duration as exerting an effect on weight through leptin-related appetitive pathways, thus raising the possibility that there are gene-sleep interactions that function via a central regulatory mechanism, such as the leptin pathway therefore conferring early risk of obesity. However, evidence from longitudinal studies would be useful to address these issues but such datasets remain scarce.

This study leverages a large sample from the Beijing Child and Adolescent Metabolic Syndrome (BCAMS) study[22] in order to test the hypothesis that sleep duration in childhood not only modifies the genetic predisposition to childhood obesity, but also has long term impact on obesity risk, as assessed at a ten-year follow-up, particularly for loci operating via the leptin pathway. Our study provides novel insight into the links between gene-environment interaction and obesity that could be targeted to reduce risk in early life.

## Subjects and methods

### Population

BCAMS was designed as an ongoing follow-up study of obesity and related metabolic abnormalities among a representative sample of school-aged children in Beijing, which has been described in detail elsewhere (Supplementary Fig. 1) [22, 23] and was registered at www.clinicaltrials.gov (NCT03421444). Briefly, the BCAMS study recruited 19,593 school children (6–18 years old) via stratified randomized sampling from four urban and three rural districts within the Beijing area between April and October 2004. Based on initial finger capillary blood tests, 4500 participants at risk of MS were identified as having one or more of the following disorders: being overweight, high blood pressures, increased total cholesterol ≥5.2 (mmol/L), triglyceride ≥1.7 (mmol/L), or fasting glucose ≥5.6 (mmol/L). Next, all children at risk of MS (*n* = 4500), together with a parallel reference sample of 1095 school children, were invited to participate in a medical examination including venipuncture-based blood sample tests. In total, 3514 participants agreed to complete further medical examination. Among them, 3211 participants (1161 without any MS component based on further examination) who had completed the baseline full examination of leptin level, genotype, and lifestyles questionnaire including sleep time were included in this study [13]. A follow-up study was conducted after 10 years of initial investigation; participants were recruited consecutively through various modalities in a center at the Beijing Chaoyang Hospital. A total of 848 participants who completed anthropometric measurements at follow-up, including 559 who completed in-depth clinical examination [23, 24], were included in the longitudinal analysis. Thus, our analysis consisted of two parts: a cross-sectional analysis using baseline data (*n* = 3211) and a prospective analysis based on follow-up data (*n* = 848), which is described in detail in Supplementary Fig. 1. Informed consent from participants and/or parents/guardians was obtained prior to entry into the study. The BCAMS study was approved by the Ethics Committee at the Capital Institute of Pediatrics in Beijing. All the phases of the study complied with the Ethical Principles for Medical Research Involving Human Subjects expressed in the Declaration of Helsinki.

### Phenotyping

The subjects’ height, weight, waist circumference, and percent body fat (FAT %) were measured according to our standard protocol [19, 22, 23]. Baseline leptin was measured by enzyme-linked immunosorbent assay with the intra- and inter-assay coefficient of variations <7.4% and <9.3%, respectively [25]. All samples were tested in duplicate and blinded. BMI was calculated as weight/height[2]. According to the Working Group for Obesity in China, age- and sex-specific BMI percentiles were used to define overweight (85th) and obesity (95th) for children and adolescents (≤18 years) [26]. For adults (>18 years), obesity was defined as BMI > 28 kg/m^2^ and overweight as BMI 24.0–28.0 kg/m [27]. The diagnosis of MS in children and adults has been described in detail elsewhere [22, 23].

### Single nucleotide polymorphism (SNP) selection and genotyping

Genomic DNA was isolated from peripheral white blood cells using the QIAamp DNA Blood Midi Kits (Qiagen). Twelve SNPs were selected from GWAS reports of obesity in East Asian ancestry populations [16–18], with particular focus on the loci harboring brain-expressed genes, such as *FTO*-rs1558902, *MC4R*-rs2331841, *BDNF*-rs2030323, *MAP2K5*-rs4776970, *GNPDA2*-rs16858082, and *PCSK1*-rs261967 (Supplementary Table 1**)**. All SNPs were genotyped on the Sequenom Mass Array iPLEX genotyping platform [28]. Repeated control samples were present in each genotyping plate, yielding a concordance rate of 100%.

### Sleep duration

Self-reported sleep duration at baseline was obtained from all participants (>12 years old) and/or their parents or guardians (≤12 years old) and was derived from the following question: “How many hours did you sleep on an average day over the past 7 days?” The response ranged from 4.5 to 13 h[13]. Sleep duration was categorized into four groups as follows: ≤7 h/day, 8 h/day, 9 h/day, and ≥10 h/day, or two groups based on the median of sleep time.

### Covariates

At baseline, pubertal development by Tanner stage of breast development in girls and testicular volume in boys was assessed by a pediatrician of the same sex as the child. Lifestyle information was collected by questionnaire. Physical activity was expressed as low (<3 times/week) or moderate-to-vigorous (≥3 times/week), meaning that each individual spent ≥30 m/activity for extracurricular physical activities (cycling, hiking, batting, running, and swimming etc.). Dietary information included ten items (breakfast, bean, seafood, milk, vegetables, fruits, red meat, sweet soft drinks, snacks, and consumption of western-fast food). The response options were represented as 1–5 depending on the degree of dietary status from “seldom or never” to “everyday” and diet scores were summed for each subject. A higher score indicated better dietary quality, and vice versa [28].

### Statistical analysis

Analyses were performed using the Statistical Package for Social Sciences (SPSS) 20.0 for Windows. Hardy–Weinberg equilibrium was assessed using the Chi-squared test in the total sample. In this study, all SNPs passed Hardy–Weinberg equilibrium. Leptin was natural logarithmically (ln) transformed before analysis due to skewed distribution. By applying Bonferroni correction, a *P*-value below 0.004 (0.05/12 SNPs) was considered significant, while a *P*-value between 0.05 and 0.004 was considered nominally significant. A score of 0, 1, or 2 was assigned to genotypes of the BMI-associated SNPs according to the number of risk alleles in an additive model. We calculated a set of genetic predisposition scores (GPS) as follow: the GPS_all_ for all 12 selected SNPs, the GPS_leptin_ for six leptin-related SNPs (Supplementary Table 1) [19]. The GPSs were calculated as the sum of risk genotypes on the basis of single SNP analyses with the additive model.

Logistic or linear regression analyses were applied to test the associations between the GPSs, sleep duration, and obesity measurement. GPS × sleep duration interaction analysis for BMI was performed using a linear regression model with SNPs coded in an additive model, including potential confounders including age, sex, residence, puberty, exercise and diet score, and sleep duration coded as a dichotomous/continuous variable. SNP × sleep duration interaction analysis for obesity and overweight was performed using logistic regression with adjustment for the same covariates. When interactions were statistically significant, stratified analyses were undertaken to observer effect modification. Because the standard definitions for sleep duration norms specific to metabolic health in Chinese population have not yet been defined. According to the 2015 US National Sleep Foundation’s recommendations [29], there are minor differences in the recommended sleep duration for youth: 9–11 h/day is recommended for children of school-age (6–13 years), and 8–10 h/day is recommended for teenagers (14–17 years). So regardless of age group, sleeping ≤7 h/day in children is not recommended and are known as short sleepers. Therefore, we classified the participants into four groups, beginning with ≤7 h/day, in order to observe the influence of every additional hour of sleep on the genetic susceptibility to childhood obesity. In addition, to exam the long term effect of sleep duration based on 10-year follow-up, we also classified the participants into two groups based on the median of sleep time (<9 h/day and ≥9 h/day) to increase the power of the analysis.

**Fig. 1:**
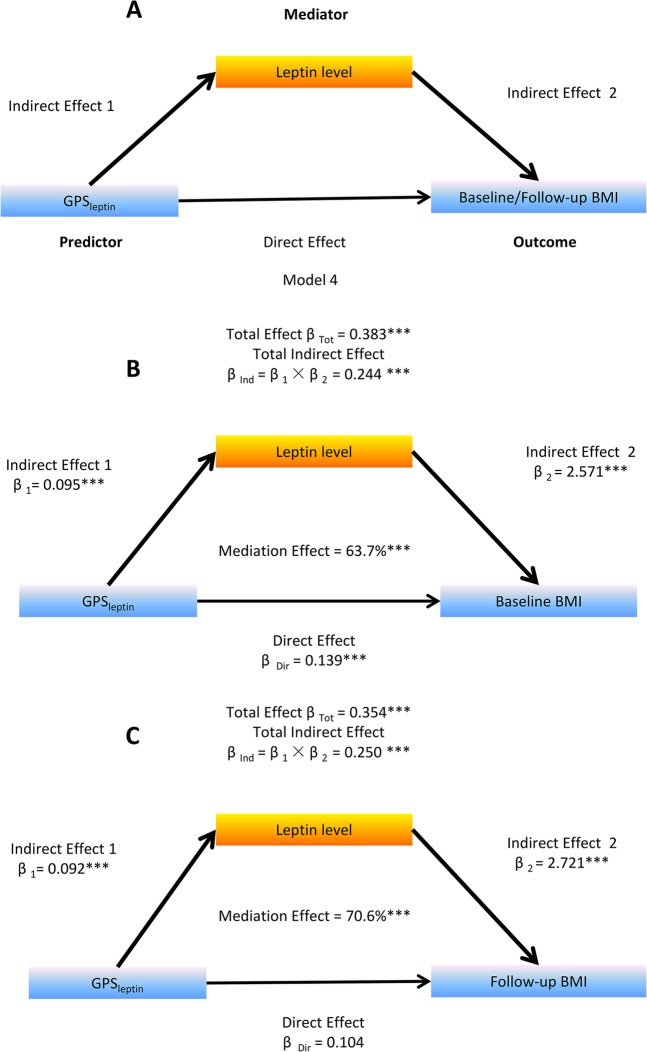
A visual schematic of the path model used to test the mediation effect of leptin on the association of GPS_leptin_ with baseline and follow-up BMI. Flow diagram showing that the mediation effect using Ln-leptin was 63.7% for baseline BMI and 70.6% for follow-up BMI. β for linear regression in the mediation models was adjusting for age, sex, residence, pubertal stages, diet score, and activity. *P *≤* 0.05, **P *≤* 0.01, ***P *≤* 0.001. BMI body mass index, GPS genetic predisposition scores

**Fig. 2:**
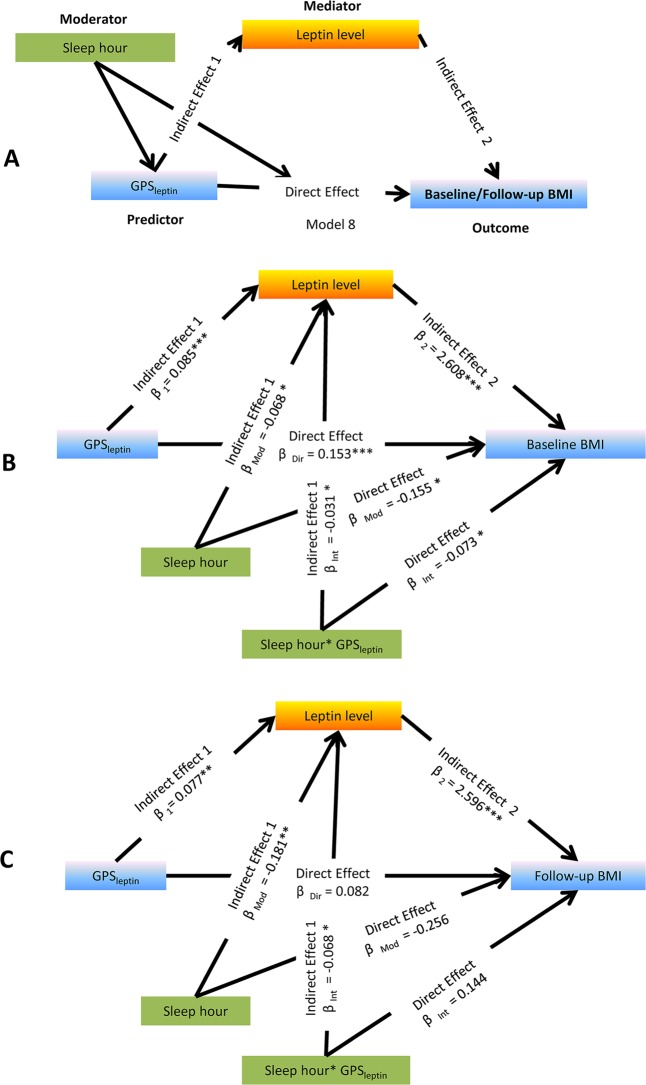
A visual schematic of the path model used to test the moderation effect of sleep duration and the mediation effect of leptin on the association of GPS_leptin_ with baseline and follow-up BMI. The proposed moderator variable was baseline sleep duration, the model was used to test for an effect of the GPS-sleep interaction on baseline and follow-up BMI; the proposed mediator variable was baseline leptin levels, where this model was used to test the paths from GPS, sleep duration, and GPS-sleep interaction respectively to Ln-leptin, and from the Ln-leptin to change in BMI at both baseline and follow-up. β for linear regression in the mediation models was adjusting for age, sex, residence, pubertal stages, diet score, and activity. *P *≤* 0.05, **P *≤* 0.01, ***P *≤* 0.001. BMI body mass index, GPS genetic predisposition scores

Mediation analysis conducted on the whole population was performed using PROCESS Procedure for SPSS Release 2.12 [30] for baseline and 10-year follow-up BMI, respectively, adjusting for baseline age, sex, Tanner stage, residence, and behavioral factors. Our previous studies have confirmed that leptin plays a mediating role, not only in the association between sleep duration and baseline BMI [13], but also in the association between genetic predisposition and baseline BMI [19]. In this study, first we extended to analyze whether leptin plays a role in mediating the prospective association between the polygene’s effect and risk of obesity at 10-year follow-up by using the template Model 4 (Fig. 1) [30]. Second, we tested whether sleep duration modifies the association between the respective polygene score and both baseline and follow-up BMI through leptin levels by using Model 8 [30]. As depicted in Fig. 2, the proposed moderator variable was baseline sleep duration, the model was used to test for an effect of the GPS-sleep interaction on baseline and follow-up BMI; the proposed mediator variable was baseline leptin levels, where this model was used to test the paths from GPS, sleep duration, and GPS-sleep interaction, respectively, to Ln-leptin, and from the Ln-leptin to change in BMI at both baseline and follow-up.

## Results

### Cohort characteristics

Baseline characteristics of the participants according to baseline and follow-up weight categories are listed in Table 1. The study was conducted in 3211 baseline and 848 follow-up unrelated individuals recruited through the BCAMS study. For baseline weight categories, 50.0% of the participants were male, and the mean age was 12.4 ± 3.1 years, and the average sleep duration was 8.5 h (median 9.0 h). Compared with the participants presenting as normal weight and overweight, a higher proportion of the children with obesity were prepubertal and male. The obesity phenotype was associated with younger age, and less frequent physical activity (all *P* *<* 0.05). Moreover, obesity-related anthropometric traits (i.e., BMI, waist circumference, and FAT %) and leptin were increased gradually from normal weight to overweight and obese groups (all *P* *<* 0.001). As expected, the significant differences in the baseline adiposity traits remained (all *P* *<* 0.001) when the participants were classified by 10-year follow-up body weight. Moreover, among the 848 participants who returned for follow-up, ~3% of the children presenting as normal weight and 12% as overweight developed obesity at 10-year follow-up, while over 85% of the children with obesity at baseline remained obese at 10-year follow-up, suggesting a well-established obesity trajectory over time from childhood to adulthood.

**Table 1:** Baseline characteristics of subjects in the three weight categories at baseline and 10-year follow-up^a^

Baseline phenotype	Baseline			*P*	10-year follow-up			*P*
	Normal weight	Overweight	Obese		Normal weight	Overweight	Obese	
Male/n (%)	582/1452 (40.1%)	289/604 (47.8%)*	734/1155 (63.5%)*^+^	**<0.001**	145/365 (39.7%)	97/191 (50.8%)*	193/292 (66.1%)*^+^	**<** **0.001**
Age (years)	12. 7 ± 3.07	13.4 ± 2.90*	11.8 ± 2.88*^+^	**<0.001**	10.4 ± 2.7	11.0 ± 2.8*	10.5 ± 2.7	**0.048**
Urban (%)	933/1452 (64.3%)	370/604 (61.3%)	718/1155 (62.2%)	0.396	243/365 (66.6%)	140/191 (73.3%)	201/292 (68.8%)	0.267
Puberty stages	2.8 ± 1.4	3.2 ± 1.4*	2.4 ± 1.4*^+^	**<0.001**	2.0 ± 1.2	2.3 ± 1.5 *	1.9 ± 1.3 ^+^	**0.012**
Diet score	35. 7 ± 4.8	35.4 ± 4.7	35.8 ± 4.8	0.199	36.9 ± 4.7	36.8 ± 4.8	36.1 ± 4.9	0.075
Physical activity ( ≥ 3 times/week) *N* (%)	848/1452 (58.4%)	326/604 (54.0%)*	631/1155 (54.6%)*	**0.008**	230/365 (63.0%)	108/191 (56.5%)	170/292 (58.2%)	0.182
BMI (kg/m^2^)	17.9 ± 2.4	23.5 ± 2.4*	26.6 ± 3.7*^+^	**<0.001**	17.7 ± 3.2	22.6 ± 3.6 *	25.2 ± 3.6 *^+^	**<** **0.001**
Waist Circumference (cm)	62.5 ± 7.1	76.3 ± 7.4*	83.6 ± 10.9*^+^	**<0.001**	60.9 ± 8.6	73.8 ± 10.6*	80.2 ± 11.2 *^+^	**<** **0.001**
Percent body fat	18.4 ± 5.7	28.2 ± 6.1*	31.0 ± 6.7*^+^	**<0.001**	18.2 ± 6.5	26.7 ± 6.8*	29.8 ± 6.2 *^+^	**<** **0.001**
Leptin (ng /ml)^b^	0.7 ± 1.1	2.1 ± 0.9*	2.6 ± 0.8*+	**<0.001**	0.8 ± 1.3	2.1 ± 1.1*	2.5 ± 0.9 *^+^	**<** **0.001**
NMW/OW/OB (%)	/	/	/	**/**	79.4%/10.7%/9.9%	20.0%/30.5%/49.5%	3.1%/12.3%/84.6%	**<** **0.001**

One-way ANOVA where differences versus normal weight subjects are indicated as *P *<* 0.05, differences versus overweight individuals are indicated as ^+^*P* *<* 0.05

Values in bold are significant at *P* < 0.05

*NMW/OW/OB* Normal weight/Overweight/Obese

^a^All values are expressed as mean ± SD, *n* (%)

^b^Skewed distributions were natural logarithmically (ln) transformed

### Sleep duration modifies the association between genetic predisposition and adiposity, both at baseline and at follow-up

Our previous cross-sectional study confirmed similar study-wide significant association with the baseline BMI and obesity for the polygene score consisting of all the 12 adult BMI loci (GPS_all_) and the GPS_leptin_ of 6 leptin-related loci [19]. In this study, we also confirmed study-wide significant association with risk of obesity and overweight at 10-year follow-up for either GPS_all_ (OR [95%CI]: 1.138 [1.066, 1.214]; *P* = 9.3 × 10^−5^) or GPS_leptin_ (1.183 [1.079, 1.296]; *P* = 3.2 × 10^−4^) (Supplementary Table 1); however, no association for GPS after excluding the six leptin-related SNPs was observed, thus further polygene analyses were restricted to GPS_leptin_.

Next, we tested for association of the six leptin-related variants and GPS_leptin_ with continuous sleep duration and dichotomized short or long sleep duration, adjusting for age, sex, and other lifestyle factors. No individual variant or GPS_leptin_ yielded significant association with sleep duration (Supplementary Table 2).

We then examined whether sleep duration modifies the effect of key variants on the risk of obesity by testing for interaction between baseline sleep duration and GPS_leptin_ in influencing BMI/obesity and overweight at both baseline (*n* = 3211) and 10-year follow-up (*n* = 848). Significant GPS_leptin_ × sleep duration interaction was observed on baseline BMI/obesity and overweight (*P*_interaction = _0.008 for BMI, *P*_interaction = _0.002 for obesity and overweight). We further conducted stratified analyses by categories of sleep duration. As shown in Table 2, for participants sleeping ≤7 h/day at baseline, after adjusting for age, sex, and other lifestyle factors, GPS_leptin_ was associated with significantly higher baseline BMI (β [95 CI%]: 0.723[0.444–1.001] kg/m^2^, *P* = 5.2 × 10^-7^) and obesity and overweight risk (OR [95%CI]: 1.328 [1.164–1.516], *P* *=* 2.6 × 10^-5^); for participants sleeping 8 h/day (*P* *=* 6.5 × 10^–4^) or 9 h/day (*P* *=* 0.003), the positive associations between GPS_leptin_ and BMI/ obesity and overweight were attenuated to a degree; however, in participants whose sleep duration ≥10 h/day, the associations were no longer significant (*P* *>* 0.05). In addition, the results were consistent when analyses were stratified by sex. At the follow-up phase, to increase the study power, we combined participants into two groups according to the median of baseline sleep duration (**<**9 h/day and ≥9 h/day). After adjusting for multiple confounders, GPS_leptin_ was significantly associated with obesity and overweight risk (OR [95%CI]: 1.300 [1.093–1.546], *P* *=* 0.003) in participants sleeping <9 h/day, but the association disappeared in participants whose sleep duration ≥9 h/day (*P* *>* 0.05). Moreover, the sleep duration could modify the effects of the *FTO*-rs1558902 risk alleles (*P* for interaction = 0.018) on BMI at baseline, while not evident in other leptin-related SNPs (Supplementary Table 3). In addition, we analyzed whether sleep duration could modify the effect of GPS_all_ on BMI/obesity and overweight and found the interaction was very similar to GPS_leptin_.

**Table 2:** Association between GPS_leptin_ and BMI/obese and Overweight according to baseline sleep duration^a^

	Variables	Sleep time ≤ 7 h/day (*n* = 518)		Sleep time = 8 h/day (*n* = 1079)		Sleep time = 9 h/day (*n* = 977)		Sleep hour ≥ 10 h/day (*n* = 637)	
		β/OR (95% CI)	*P*	β/OR (95% CI)	*P*	β/OR (95% CI)	*P*	β/OR (95% CI)	*P*
Baseline	BMI (kg^.^m^**−**2^)								
	Model 1	0.736 (0.472**−**1.000)	**7.2E−8**	0.346 (0.162–0.529)	**2.3E−4**	0.291 (0.109**−**0.474)	**0.002**	0.245 (0.025**−**0.464)	0.029
	Model 2	0.723 (0.444**−**1.001)	**5.2E−7**	0.326 (0.139–0.513)	**6.5E−4**	0.276 (0.094**−**0.457)	**0.003**	0.219 (**−**0.004**−**0.442)	0.055
	Obese and overweight/lean								
	Model 1	1.313 (1.161–1.484)	**1.4E−05**	1.209 (1.111**−**1.317)	**1.2E−5**	1.169 (1.070**−**1.277)	**0.001**	1.092 (0.985**−**1.212)	0.096
	Model 2	1.328 (1.164–1.516)	**2.6E−05**	1.213 (1.109**−**1.325)	**2.2E−5**	1.175 (1.075**−**1.294)	**6.0E-4**	1.088 (0.975**−**1.214)	0.131
				Sleep time < median (9 h /day) (*n* = 312)		Sleep time ≥ median (9 h/day) (*n* = 536)			
				β/OR (95% CI)	*P*	β/OR (95% CI)	*P*		
Follow-up	BMI (kg.m^−2^)								
	Model 1			0.473 (0.075**−**0.871)	0.020	0.190 (−0.124–0.503)	0.235		
	Model 2			0.458 (0.066**−**0.849)	0.022	0.190 (−0.124–0.503)	0.235		
	Obese and overweight/lean								
	Model 1			1.324 (1.120**−**1.564)	**0.001**	1.108 (0.990–1.239)	0.073		
	Model 2			1.300 (1.093**−**1.546)	**0.003**	1.106 (0.984–1.2440	0.092		

Model 1 adjusted for age and sex; model 2 further adjusted for residence, puberty, exercise, and diet score based on model 1

The mean sleep hour was 8.5 h/day

Values in bold are significant at *P* < 0.004 after Bonferroni correction

*GPS* genetic predisposition scores

^a^All values are expressed as β/OR (95% CI). β/OR (95% CI) and P value for linear and logistic regression in the additive model

### Leptin mediated the association between genetic predisposition and adiposity

To exam whether leptin mediates the effect of polygenic risk on obesity from childhood to young adulthood, we tested for mediation effect of baseline leptin on GPS_leptin_–BMI associations adjusting for baseline age, sex, residence, pubertal stages, and other lifestyle factors. As listed in Fig. 1, the total effect of GPS_leptin_ on baseline (β_Total_ = 0.383, *P* *<* 0.001) and 10-year follow-up BMI (β_Total_ = 0.354, *P* *<* 0.001) were estimated without leptin in the models. The total indirect effect via leptin defined as the product of indirect effect 1 (β_1_) and indirect effect 2 (β_2_) was significant at both baseline (β _Indirect_ = 0.244, *P* *<* 0.001) and follow-up BMI (β _Indirect_ = 0.250, *P* *<* 0.001). The net mediation effects of leptin on baseline and follow-up BMI were 63.7% and 70.6%, respectively.

### Moderation of sleep duration and mediation of leptin on the association between genetic predisposition and adiposity

We sought to test for both the moderation of sleep duration and the mediation of leptin on polygenic risk influencing BMI with a long-term effect (visual schematic of the path model shown in Fig. 2). At baseline, we observed nominally significant interactions between GPS_leptin_ and sleep duration for BMI and leptin concentrations, with a 0.073 kg/m^2^ lower BMI and 0.031 ng/ml lower Ln-leptin with each additional sleeping hour per risk allele, and the total indirect mediation effect of baseline leptin (β = –0.081 kg/m^2^, *P* *<* 0.05) on the GPS_leptin_-childhood BMI association was 52.6%. Meanwhile, longer sleep duration weakened the effect of GPS_leptin_ on leptin at 10-year follow-up, with a 0.068 ng/ml lower Ln-leptin with each additional sleeping hour per risk allele, while there was a significant indirect effect of the interactions of GPS_leptin_ and sleep duration on 10-year follow-up BMI (β = -0.177, *P* *<* 0.05); however, the mediated effect of leptin was not in the same direction as the direct effect, thus the proportion mediated was undefined for follow-up BMI. Table 3 summarizes the conditional direct and indirect effects of GPS_leptin_ on baseline and 10-year follow-up BMI at the mean values ±1 SD of the baseline sleep duration. The moderation effect of sleep duration yielded a significant direct effect of 0.153 kg/m^2^ and indirect effect of 0.223 kg/m^2^ (all *P* *<* 0.05) on the baseline BMI, while only appeared a significant indirect effect of 0.199 kg/m^2^ (*P* *<* 0.05) on the follow-up BMI. Meanwhile, both the direct and indirect effects were attenuated with the increase in sleep duration, with the finding indicating that sleep duration modified the polygenic obesity risk mainly via the leptin pathway to impact 10-year follow-up BMI.

**Table 3:** Direct and indirect effects of GPS_leptin_ on baseline and 10-year follow-up BMI at different values of the sleep duration at baseline^a^

Baseline BMI						Follow-up BMI			
*Conditional* ***direct*** *effects of GPS*_*leptin*_ *on baseline and 10-year follow-up BMI at values of the sleep duration:*									
*Sleep duration*	*Effect*	*SE*	*95% CI*	*P*	*Sleep duration*	*Effect*	*SE*	*95%CI*	*P*
−1 SD (1.0 h)	**0.224**	**0.050**	**0.126−0.322**	**7.5E−6**	**−**1 SD (1.0 h)	**−**0.040	0.142	**−**0.319**−**–0.239	0.778
Mean (8.5 h)	**0.153**	**0.036**	**0.083−0.223**	**1.8E−5**	Mean (8.5 h)	0.082	0.101	**−**0.116**−**0.280	0.416
+1 SD (1.0 h)	0.082	0.050	−0.0162–0.180	0.102	+1 SD (1.0 h)	0.204	0.143	**−**0.077**−**0.485	0.154
*Conditional* ***indirect*** *effects of GPS*_*leptin*_ *on baseline and 10-year follow-up BMI at values of the sleep duration: through Ln-leptin*									
*Sleep duration*	*Effect*	*SE*	*95% CI*		*Sleep duration*	*Effect*	*SE*	*95%CI*	
−1 SD (1.0 h)	**0.302**	**0.049**	**0.203−0.395**	**<0.05**	−1 SD (1.0 h)	**0.348**	**0.096**	**0.165–0.544**	**<0.05**
Mean (8.5 h)	**0.223**	**0.038**	**0.148−0.301**	**<0.05**	Mean (8.5 h)	**0.199**	**0.075**	**0.051–0.348**	**<0.05**
+ 1 SD (1.0 h)	**0.143**	**0.057**	**0.035−0.257**	**<0.05**	+1 SD (1.0 h)	0.049	0.112	−0.174–0.267	>0.05

*β* (95% CI) and *P* value for linear regression in the mediation models

*P* value was adjusting for age, sex, residence, pubertal stages, diet score, and activity at baseline

Values for moderators i.e., sleep durations are the mean and ±1 SD from mean

Values in bold are significant at *P* < 0.05

*GPS* genetic predisposition scores, *BMI* body mass index

^a^The data table shows coefficient estimates for direct and indirect effects of GPS_leptin_ on baseline and 10-year follow-up BMI at values of sleep duration at baseline

## Discussion

In this cohort study of youth from China, we report evidence for interaction between sleep duration and genetic factors in the modulation of body weight at baseline and at 10-year follow-up. The most notable finding is that the interaction effect of the polygene risk score and baseline sleep duration on BMI was significantly mediated by elevated leptin concentration. Therefore, our results indicate that adequate sleep time in childhood appears to reverse the adverse effects of BMI-related loci on obesity from childhood to adulthood, particularly through the leptin pathway.

Our results confirm and extend the association between self-reported short sleep duration and risk of obesity observed in previous studies, both in adult [31–33] and in children [34, 35]. Meanwhile, the central nervous system (CNS), especially the hypothalamus is currently considered to be primarily responsible for controlling appetite and body weight. Sleep disturbances can influence brain functions involved in appetite and energy balance regulation, which are related to obesity, and higher risk of cardio-metabolic disorders [31, 33, 36]. Notably, the brain-expressed genes located at key BMI-related loci, such as *FTO, MC4R, MAP2K5, GNPDA2, PCSK1*, and *BDNF*, may yield many connections with sleep duration. As far as we are aware, the only previous study reporting an *FTO*-sleep duration association found evidence that TT homozygotes (but not A* carriers) for the *FTO* SNP rs9939609 (in high LD with the SNP we studied, rs1558902, *r*^2^_CEU_ = 0.922) was nominally associated with decreasing sleep duration in Caucasian children aged 7 [37]. A role for *BDNF* in sleep homeostasis has also been proposed [38–40]. *BDNF* mRNA levels are higher after wakefulness and BDNF levels were positively correlated with the duration of the following sleep period [38, 39]. However, no data is available regarding the relationships between the other four genes (*MC4R, MAP2K5, GNPDA2*, and *PCSK1*) and sleep duration. In this study, we observed no significant associations between sleep duration and the six leptin-related SNPs and GPS_leptin_, indicating that sleep duration may act as a modifier rather than a mediator in the association between genetic risk and pediatric BMI.

As previous authors have argued, shorter sleep duration (sleep <7 h/day) increases the expression of genetic risks for high BMI in adults from the US (mean 36.6 years) [20], implying that the genetic effects on BMI differ depending on sleep. Recently, Young et al. reported that squared deviations from mean sleep duration (7.18 h) are associated with an enhanced effect of *FTO-*rs1421085 on adult (mean 56.8 years) BMI [21]. In this study, first, we found that short sleep duration in childhood enhanced the effect of *FTO-*rs1558902 on pediatric BMI but not with BMI of grown-ups (likely due to less study power in follow-up population). Second, sleep duration significantly mediated the association between adiposity and polygenic obesity risk score (GPS_leptin_) that excluded *FTO* both in childhood and adulthood (*P* *<* 0.05), thus, the associations observed in our study were not explained entirely by variant residing in *FTO* (data not shown). Our finding supports the hypothesis that one variant contribute only to a small degree, while additive polygenic SNP models have a more significant effect on common obesity.

Notably, we found that childhood sleep duration significantly modulated the association between polygenic obesity risk score (GPS_leptin_) and pediatric and the 10-year follow-up BMI. This suggests that inadequate sleep time in early life has long-term adverse impact on genetically susceptible individuals through the development of obesity.

However, the mechanisms by which inadequate sleep increases the genetic risk effect on obesity pathogenesis are poorly understood. Currently, adipose tissue has been recognized as a dynamic endocrine organ, producing a range of biologically active substances, collectively called “adipokines”, which regulate energy homeostasis, inflammation, insulin resistance, and cardiovascular function [41]. Among the adipokines, leptin is the most thoroughly examined in the literature, and is recognized as a fundamental regulator of energy homeostasis, acting through the binding of its functional long isoform leptin receptor in the CNS to control feeding behavior and energy expenditure, and modulating glucose/lipid metabolism in peripheral tissues [42]. However, there is an ongoing debate on the association between sleep duration and leptin levels [14]. Previous studies have revealed that leptin levels are variable with short sleep duration, either increased [9, 11, 14] or decreased [8, 14]. In our recent cohort study of children with risk for MS [13], we have reported that sleep duration was negatively associated with circulating leptin levels even after adjustment for BMI. Given our cohort consists of a high proportion of participants with overweight and obesity (54.8%), among whom hyperleptinemia or leptin resistance were relatively common, our finding can be interpreted as chronic lack of sleep reducing leptin sensitivity; thus, an elevation in circulating leptin may reflect a secondary increase in an attempt to overcome this leptin resistance. In addition, we also found that leptin could mediate the association between sleep duration and cardiometabolic risk factors, and accounted for 81.6% of the total effect of sleep duration on BMI [13]. Based on our current analysis, it is likely that the childhood sleeping time acts as a long term modifier on the effect of polygenic risk for obesity through the leptin pathway.

Among the six leptin-associated CNS-highly-expressed loci, three genes (*MC4R, BDNF*, and *PCSK1*) are known to be involved in the hypothalamic leptin–melanocortin pathway [15, 43, 44]. *FTO* is the first GWAS-identified obesity-susceptibility locus and shows the strongest association with BMI in our pediatric cohort [19]. Unlike the above-noted three genes with possible function through hypothalamic leptin–melanocortin pathway, FTO modulates leptin receptor localization within neurons to control food intake and adiposity [45]. However, there is much debate on which is the actual effector gene(s) at this locus. Furthermore, *MAP2K5* and *GNPDA2* are also highly-expressed in CNS and positive correlated with leptin [19]. Given the fact that the genes at these six loci are parts or have well-established connections with leptin in literature, it is not surprising that we found the modifying effect of sleep duration partly via leptin.

On the other hand, we analyzed the modification of sleep duration on the effect of GPS_all_ (all the 12 selected SNPs) and GPS_leptin-unrelated_ (six leptin-unrelated SNPs) on BMI and leptin. However, we observed no significant interactions between GPS_all_, GPS_leptin-unrelated_, and sleep duration on baseline concentrations of leptin (data not shown). The results further confirmed our hypothesis that sleep duration modifies the effect of GPS_leptin_ on obesity to a degree via the leptin pathway.

This study has a number of strengths. This is the first longitudinal study to quantify the modification of sleep duration on polygenic obesity risk score and explore the possible mechanisms for their impact on BMI, providing novel intervention targets in early life. In addition, analyzing a large, well-characterized cohort of participants with a range of covariates makes our results more robust by adjusting for many potential confounders known to be associated with obesity. Nevertheless, there are several limitations that should be noted. First, mainly due to great migration to other parts of the country or to other countries as those school-aged children growing up with the rapid development of society and economy in Beijing, the number of participants returning to follow-up after 10 years was relatively small compared with our original population at baseline. However, this longitudinal cohort enables polygenic obesity risk assessment, providing sufficient power (83%) to detect a significant causal association with our sample size of 848 participants. Second, we conducted a large longitudinal cohort study for the possible sleep-gene interaction for obesity risk, our results are considered as a credible reference for the children at risk of MS, however, it is not clear whether these results are further generalizable to all children. Third, our findings were obtained from participants from China exclusively and therefore cannot be generalized to other ethnic populations without further replication. In addition, participants in our study have a large age span at baseline, especially have a small number at follow-ups; due to the relatively small sample size after age stratification, a larger sample size will be needed ultimately to explore the effects of sleep time on the risk of future obesity in different childhood age stratification. Moreover, the habitual sleep duration is recorded through self-report questionnaires rather than by laboratory examination, and therefore, may influence the accuracy of the data. However, the advantage of using such approach is the cost-effectiveness in the context of large sample size. Previous studies, especially for those with large sample sizes, they also comparably leveraged habitual sleep time data via a questionnaire approach [8–10]. Finally, there is a scarcity of information on the multiple sleep dimensions, e.g., patterns and quality.

In summary, our longitudinal study supports the notion that increased sleep time during childhood has advantageous effects on protecting against a genetic predisposition to obesity, with leptin playing a key role in the process. Therefore, we recommend getting adequate sleep in childhood as an important intervention to prevent obesity, especially if there is a genetic predisposition to this trait. As these are among the only longitudinal data on the interaction between sleep duration and polygenic factors in the modulation of body weight, further prospective studies with larger sample size from diverse pediatric populations are warranted to further validate our findings.

## Supplementary information

Supplementary figure legend

Supplementary Figure 1. Flow diagram of the BCAMS study

Text summary

Supplementary Table 1. Association of the individual candidate SNPs with baseline and 10-year follow-up BMI/Obesity & Overweight

Supplementary Table 2. Association of sleep duration and selected SNPs/GPS

Supplementary Table 3. Association between six leptin-related SNPs and obesity-measures at baseline according sleep duration
