# Supplementary material for: Childhood sleep duration modifies the polygenic risk for obesity in youth through leptin pathway: the Beijing Child and Adolescent Metabolic Syndrome cohort study
Source: Int J Obes (Lond). 2019 Jul 8;43(8):1556–67. doi: 10.1038/s41366-019-0405-1 (PMC6760591; doi:10.1038/s41366-019-0405-1)
Supplement: Supplementary file 1 — Supplementary figure legend [file 41366_2019_405_MOESM1_ESM.docx]

**Supplementary Figure Legend**

**Supplementary Figure 1. Flow diagram of the BCAMS study**

The Beijing Child and Adolescent Metabolic Syndrome study (BCAMS) study recruited 19,593 school children (6-18 years old) via stratified randomized sampling from 4 of these urban and 3 rural districts within in Beijing area. Based on initial finger capillary blood tests, 4,500 subjects at risk for metabolic syndrome were identified as having one or more of the following disorders: being overweight, high blood pressures (> 90th percentile), increased total cholesterol ≥ 5.2 (mmol/L), triglyceride ≥ 1.7 (mmol/L) or fasting glucose ≥ 5.6 (mmol/L). Next, all children at risk of metabolic syndrome (n = 4,500), together with a parallel reference population of 1,095 schoolchildren, were invited to participant further in medical examination. Finally, 3,211 subjects completed the baseline full examination of leptin level, genotype and lifestyles questionnaire (Normal weight: n = 1,452; Overweight&obese: n = 1,759); after 10 years of initial investigation, a total of 848 subjects completed the anthropometric measurements (Normal weight: n = 365; Overweight&obese: n = 483).

: Indicating sample source.
