# Supplementary figures and images for: Childhood sleep duration modifies the polygenic risk for obesity in youth through leptin pathway: the Beijing Child and Adolescent Metabolic Syndrome cohort study
Source: Int J Obes (Lond). 2019 Jul 8;43(8):1556–67. doi: 10.1038/s41366-019-0405-1 (PMC6760591; doi:10.1038/s41366-019-0405-1)

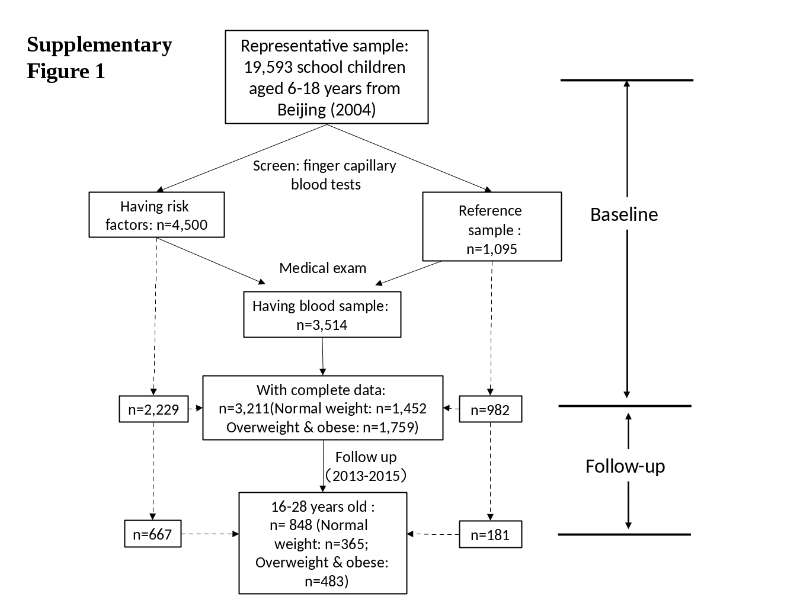

Supplement: Supplementary file 2 — Supplementary Figure 1. Flow diagram of the BCAMS study [file 41366_2019_405_MOESM2_ESM.jpg]
