# Supplementary material for: Childhood sleep duration modifies the polygenic risk for obesity in youth through leptin pathway: the Beijing Child and Adolescent Metabolic Syndrome cohort study
Source: Int J Obes (Lond). 2019 Jul 8;43(8):1556–67. doi: 10.1038/s41366-019-0405-1 (PMC6760591; doi:10.1038/s41366-019-0405-1)
Supplement: Supplementary file 3 — Text summary [file 41366_2019_405_MOESM3_ESM.docx]

Text summary

Supplementary Table 1. Association of the individual candidate SNPs with baseline and 10-year follow-up BMI/Obesity & Overweight

Supplementary Table 2. Association of sleep duration and selected SNPs/GPS

Supplementary Table 3. Association between six leptin-related SNPs and obesity-measures at baseline according sleep duration
