# Supplementary material for: Childhood sleep duration modifies the polygenic risk for obesity in youth through leptin pathway: the Beijing Child and Adolescent Metabolic Syndrome cohort study
Source: Int J Obes (Lond). 2019 Jul 8;43(8):1556–67. doi: 10.1038/s41366-019-0405-1 (PMC6760591; doi:10.1038/s41366-019-0405-1)
Supplement: Supplementary file 4 — Supplementary Table 1. Association of the individual candidate SNPs with baseline and 10-year follow-up BMI/Obesity & Overweight [file 41366_2019_405_MOESM4_ESM.docx]

| Supplementary Table 1. Association of the individual candidate SNPs with baseline and 10-year follow-up BMI/Obesity & Overweight | | | | | | | | |
| --- | --- | --- | --- | --- | --- | --- | --- | --- |
| SNPs | Nearest gene | a/A^1^ |  | *P* (HWE)^3^ | *P^4^* | | | |
|  |  |  | MAF^2^ |  | Baseline  Obesity & Overweight | Follow-up  Obesity & Overweight | Baseline  BMI | Follow-up  BMI |
| rs1558902 | *FTO* | T/A | 0.10 | 0.18 | **1.6E-5** | 0.026 | **4.6E-7** | 0.027 |
| rs2331841 | *MC4R* | A/G | 0.22 | 0.59 | **0.001** | 0.093 | **1.3E-4** | 0.393 |
| rs16858082 | *GNPDA2* | C/T | 0.34 | 0.21 | **1.7E-4** | 0.073 | **0.001** | 0.016 |
| rs261967 | *PCSK1* | A/C | 0.41 | 0.97 | **0.003** | 0.225 | 0.005 | 0.995 |
| rs4776970 | *MAP2K5* | T/A | 0.22 | 0.10 | **0.001** | 0.014 | 0.006 | 0.015 |
| rs2030323 | *BDNF* | T/G | 0.47 | 0.88 | 0.030 | 0.942 | 0.046 | 0.888 |
| *GPS_leptin_^5^* | | / | / | / | **2.5E-14** | **3.2E-4** | **1.4E-11** | **0.003** |
| rs516636 | *SEC16B* | C/A | 0.20 | 0.55 | **0.001** | 0.390 | 0.005 | 0.478 |
| rs2535633 | *ITIH4* | C/G | 0.41 | 0.47 | 0.024 | 0.018 | 0.044 | 0.025 |
| rs6545814 | *ADCY3/RBJ* | A/G | 0.41 | 0.93 | 0.175 | 0.145 | 0.047 | 0.472 |
| rs652722 | *PAX6* | T/C | 0.34 | 0.87 | 0.219 | 0.713 | 0.303 | 0.851 |
| rs12597579 | *GP2* | T/C | 0.28 | 0.64 | 0.395 | 0.531 | 0.252 | 0.771 |
| rs2237892 | *KCNQ1* | T/C | 0.31 | 0.11 | 0.737 | 0.623 | 0.655 | 0.404 |
| *GPS_all_^6^* | | / | / | / | **9.1E-13** | **9.3E-5** | **9.4E-14** | **0.001** |

^1^a: non-effect; A: effect.

^2^ Minor allele frequency.

^3^*P* value for Hardy-Weinberg equilibrium test.

*^4^P* value for logistic/linear regression in the additive model were adjusted for sex, age, Tanner stage and residence.

^5^Genetic predisposition score for *FTO*-rs1558902, *MC4R*-rs2331841, *BDNF*-rs2030323, *MAP2K5*-rs4776970, *GNPDA2*-rs16858082 and *PCSK1*-rs261967.

^6^Genetic predisposition score for *FTO*-rs1558902, *MC4R*-rs2331841, *BDNF*-rs2030323, *MAP2K5*-rs4776970, *GNPDA2*-rs16858082, *PCSK1*-rs261967, *SEC16B-* rs516636, *ITIH4-* rs2535633, *ADCY3/RBJ-* rs6545814, *PAX6-* rs652722, *GP2-* rs12597579 and *KCNQ1-* rs2237892.

Note: Values in bold are significant at *P* < 0.004 after [Bonferroni](https://www.bing.com/search?q=bonferroni+adjusted+p+value&FORM=QSRE1) correction.

SNP, Single nucleotide polymorphism; *ADCY3/RBJ,* adenylate cyclase 3; *BDNF*, brain-derived neurotrophic factor; *FTO*, fat mass and obesity associated; *GP2*, glycoprotein 2; *GNPDA2*, glucosamine-6-phosphate deaminase 2; *ITIH4*, inter-alpha-trypsin inhibitor heavy chain family, member 4; *KCNQ1*, potassium channel, voltage gated KQT-like subfamily Q, member 1; *MAP2K5*, mitogen-activated protein kinase 5; *MC4R*, melanocortin 4 receptor; *PAX6*, paired box 6; *PCSK1*, proprotein convertase subtilisin/kexin type 1; *SEC16B*, SEC16 homolog B, endoplasmic reticulum export factor.
