# Supplementary material for: Childhood sleep duration modifies the polygenic risk for obesity in youth through leptin pathway: the Beijing Child and Adolescent Metabolic Syndrome cohort study
Source: Int J Obes (Lond). 2019 Jul 8;43(8):1556–67. doi: 10.1038/s41366-019-0405-1 (PMC6760591; doi:10.1038/s41366-019-0405-1)
Supplement: Supplementary file 5 — Supplementary Table 2. Association of sleep duration and selected SNPs/GPS [file 41366_2019_405_MOESM5_ESM.docx]

| Supplementary Table 2. Association of sleep duration and selected SNPs/GPS | | | | | |
| --- | --- | --- | --- | --- | --- |
| SNP | Nearest gene | Sleep hour | | Sleep group | |
|  |  | β (95%CI) | *P* | OR (95%CI) | *P* |
| rs1558902 | *FTO* | -0.016 (-0.093-0.061) | 0.692 | 0.976 (0.815 1.169) | 0.793 |
| rs2331841 | *MC4R* | 0.013 (-0.046-0.072) | 0.661 | 1.057 (0.921-1.214) | 0.429 |
| rs16858082 | *GNPDA2* | 0.020 (-0.033-0.072) | 0.462 | 1.011 (0.894-1.142) | 0.863 |
| rs261967 | *PCSK1* | -0.011 (-0.063-0.040) | 0.668 | 0.993 (0.881-1.120) | 0.909 |
| rs4776970 | *MAP2K5* | 0.010 (-0.049-0.069) | 0.738 | 0.948 (0.828-1.086) | 0.444 |
| rs2030323 | *BDNF* | -0.033 (-0.085-0.018) | 0.200 | 0.945 (0.839-1.065) | 0.353 |
| *GPS_leptin_^1^* | | 0.000 (-0.023-0.024) | 0.976 | 0.997 (0.944-1.053) | 0.905 |

^1^ Genetic predisposition score for *FTO*-rs1558902, *MC4R*-rs2331841, *BDNF*-rs2030323, *MAP2K5*-rs4776970, *GNPDA2*-rs16858082 and *PCSK1*-rs261967.

SNP, Single nucleotide polymorphism; GPS, Genetic predisposition scores; BDNF, brain-derived neurotrophic factor; *FTO*, fat mass and obesity associated; *GNPDA2*, glucosamine-6-phosphate deaminase 2; *MAP2K5*, mitogen-activated protein kinase 5; *MC4R*, melanocortin 4 receptor; *PCSK1*, proprotein convertase subtilisin/kexin type 1.
