# Supplementary material for: Childhood sleep duration modifies the polygenic risk for obesity in youth through leptin pathway: the Beijing Child and Adolescent Metabolic Syndrome cohort study
Source: Int J Obes (Lond). 2019 Jul 8;43(8):1556–67. doi: 10.1038/s41366-019-0405-1 (PMC6760591; doi:10.1038/s41366-019-0405-1)
Supplement: Supplementary file 6 — Supplementary Table 3. Association between six leptin-related SNPs and obesity-measures at baseline according sleep duration [file 41366_2019_405_MOESM6_ESM.docx]

| \| Supplementary Table 3. Association between six leptin-related SNPs and obesity-measures at baseline according sleep duration*^1^* \| \| \| \| \| \| \| \| \| \| \| \| --- \| --- \| --- \| --- \| --- \| --- \| --- \| --- \| --- \| --- \| --- \| \| Gene \| SNP \| Variables \| Sleep hour ≤ 7 (n = 518) \| \| Sleep hour = 8 (n = 1079) \| \| Sleep hour = 9 (n = 977) \| \| Sleep hour ≥ 10 (n = 637) \| \| \| *β* (95% CI) \| *P* \| *β* (95% CI) \| *P* \| *β* (95% CI) \| *P* \| *β* (95% CI) \| *P* \| \| *FTO* \| rs1558902 \| BMI (kg.m^-2^)  *P for interaction =* ***0.018*** \| \| \| \| \| \| \| \| \| \| Model 1 \| 2.192 (1.271-3.113) \| **3.8E-6** \| 0.799 (0.215-1.384) \| 0.007 \| 0.524 (-0.059-1.108) \| 0.078 \| 0.745 (-0.014-1.504) \| 0.054 \| \| Model 2 \| 2.065 (1.096-3.035) \| **3.5E-5** \| 0.797 (0.201-1.394) \| 0.009 \| 0.450 (-0.133-1.034) \| 0.130 \| 0.860 (0.088-1.632) \| 0.029 \| \| *MC4R* \| rs2331841 \| BMI (kg/m^-2^) \| *P for interaction = 0.993* \| \| \| \| \| \| \| \| \| Model 1 \| 1.294 (0.614-1.974) \| **2.1E-4** \| 0.082 (-0.385-0.550) \| 0.729 \| 0.416 (-0.046-0.878) \| 0.077 \| 0.748 (0.201-1.295) \| 0.007 \| \| Model 2 \| 0.978 (0.256-1.700) \| 0.008 \| 0.026 (-0.451-0.504) \| 0.914 \| 0.378 (-0.083-0.839) \| 0.108 \| 0.777 (0.221-1.333) \| 0.006 \| \| *PCSK1* \| rs261967 \| BMI (kg/m^-2^*) P for interaction = 0.228* \| \| \| \| \| \| \| \| \| \| Model 1 \| 0.381 (-0.250-1.012) \| 0.236 \| 0.343 (-0.057-0.743) \| 0.093 \| 0.182 (-0.218-0.583) \| 0.372 \| 0.146 (-0.340-0.631) \| 0.556 \| \| Model 2 \| 0.510 (-0.158-1.179) \| 0.134 \| 0.327 (-0.080-0.735) \| 0.115 \| 0.084 (-0.318-0.487) \| 0.681 \| 0.151 (-0.341-0.643) \| 0.547 \| \| *MAP2K5* \| rs4776970 \| BMI (kg.m^-2^) *P for interaction = 0.185* \| \| \| \| \| \| \| \| \| \| Model 1 \| 0.594 (–0.075-1.264) \| 0.082 \| 0.356 (-0.097-0.810) \| 0.123 \| 0.516 (0.058-0.975) \| 0.027 \| -0.165 (-0.735-0.405) \| 0.57 \| \| Model 2 \| 0.518 (-0.185-1.221) \| 0.148 \| 0.363 (-0.099-0.825) \| 0.124 \| 0.526 (0.069-0.984) \| 0.024 \| -0.140 (-0.718-0.438) \| 0.634 \| \| *GNPDA2* \| rs16858082 \| BMI (kg.m^-2^*) P for interaction = 0.144* \| \| \| \| \| \| \| \| \| \| \| Model 1 \| 0.525 (-0.087-1.137) \| 0.093 \| 0.450 (0.041-0.859) \| 0.031 \| 0.172 (-0.241-0.586) \| 0.413 \| 0.187 (-0.311-0.685) \| 0.462 \| \| Model 2 \| 0.578 (-0.065-1.221) \| 0.078 \| 0.427 (0.010-0.844) \| 0.045 \| 0.314 (-0.100-0.728) \| 0.136 \| 0.081 (-0.430-0.591) \| 0.757 \| \| *BDNF* \| rs2030323 \| BMI (kg.m^-2^) *P for interaction = 0.237* \| \| \| \| \| \| \| \| \| \| Model 1 \| 0.469 (-0.134-1.073) \| 0.127 \| 0.170 (-0.230-0.570) \| 0.405 \| 0.206 (-0.191-0.604) \| 0.308 \| -0.012 (-0.497-0.472) \| 0.961 \| \| Model 2 \| 0.574 (-0.059-1.207) \| 0.075 \| 0.179 (-0.228-0.586) \| 0.388 \| 0.164 (-0.234-0.561) \| 0.420 \| -0.093 (-0.587-0.400) \| 0.710 \| |
| --- | --- | --- | --- | --- | --- | --- | --- | --- | --- | --- | --- | --- | --- | --- | --- | --- | --- | --- | --- | --- | --- | --- | --- | --- | --- | --- | --- | --- | --- | --- | --- | --- | --- | --- | --- | --- | --- | --- | --- | --- | --- | --- | --- | --- | --- | --- | --- | --- | --- | --- | --- | --- | --- | --- | --- | --- | --- | --- | --- | --- | --- | --- | --- | --- | --- | --- | --- | --- | --- | --- | --- | --- | --- | --- | --- | --- | --- | --- | --- | --- | --- | --- | --- | --- | --- | --- | --- | --- | --- | --- | --- | --- | --- | --- | --- | --- | --- | --- | --- | --- | --- | --- | --- | --- | --- | --- | --- | --- | --- | --- | --- | --- | --- | --- | --- | --- | --- | --- | --- | --- | --- | --- | --- | --- | --- | --- | --- | --- | --- | --- | --- | --- | --- | --- | --- | --- | --- | --- | --- | --- | --- | --- | --- | --- | --- | --- | --- | --- | --- | --- | --- | --- | --- | --- | --- | --- | --- | --- | --- | --- | --- | --- | --- | --- | --- | --- | --- | --- | --- | --- | --- | --- | --- | --- | --- | --- | --- | --- | --- | --- | --- | --- | --- | --- | --- | --- | --- | --- | --- | --- | --- | --- | --- | --- | --- | --- | --- | --- | --- | --- | --- | --- | --- | --- | --- |

*^1^β* (95% CI) and *P* value for linear regression in the additive model.

Model 1 adjusted for age and sex; model 2 further adjusted for residence, puberty, exercise and diet score based on model 1.

Values in bold are significant at *P* < 0.004 after [Bonferroni](https://www.bing.com/search?q=bonferroni+adjusted+p+value&FORM=QSRE1) correction.

SNP, Single nucleotide polymorphism; *BDNF*, brain-derived neurotrophic factor; *FTO*, fat mass and obesity associated; *GNPDA2*, glucosamine-6-phosphate deaminase 2; *MAP2K5*, mitogen-activated protein kinase 5; *MC4R*, melanocortin 4 receptor; *PCSK1*, proprotein convertase subtilisin/kexin type 1.
